# Human Plasma Transcriptome Implicates Dysregulated S100A12 Expression: A Strong, Early-Stage Prognostic Factor in ST-Segment Elevated Myocardial Infarction: Bioinformatics Analysis and Experimental Verification

**DOI:** 10.3389/fcvm.2022.874436

**Published:** 2022-06-01

**Authors:** Hu Zhai, Lei Huang, Yijie Gong, Yingwu Liu, Yu Wang, Bojiang Liu, Xiandong Li, Chunyan Peng, Tong Li

**Affiliations:** ^1^Department of Heart Center, The Tianjin Third Central Hospital, Tianjin, China; ^2^Tianjin Key Laboratory of Extracorporeal Life Support for Critical Diseases, Tianjin, China; ^3^Artificial Cell Engineering Technology Research Center, Tianjin, China; ^4^Tianjin Institute of Hepatobiliary Disease, Tianjin, China; ^5^The Third Central Clinical College, Tianjin Medical University, Tianjin, China; ^6^Department of Laboratory Medicine, Taihe Hospital, Hubei University of Medicine, Shiyan, China; ^7^Hubei Key Laboratory of Wudang Local Chinese Medicine Research, Hubei University of Medicine, Shiyan, China; ^8^Hubei Key Laboratory of Embryonic Stem Cell Research, Hubei University of Medicine, Shiyan, China

**Keywords:** acute myocardial infarction, differentially expressed genes, microarray analysis, transcriptome, prognostic biomarker, bioinformatics

## Abstract

The ability of blood transcriptome analysis to identify dysregulated pathways and outcome-related genes following myocardial infarction remains unknown. Two gene expression datasets (GSE60993 and GSE61144) were downloaded from Gene Expression Omnibus (GEO) Datasets to identify altered plasma transcriptomes in patients with ST-segment elevated myocardial infarction (STEMI) undergoing primary percutaneous coronary intervention. GEO2R, Gene Ontology/Kyoto Encyclopedia of Genes and Genomes annotations, protein–protein interaction analysis, etc., were adopted to determine functional roles and regulatory networks of differentially expressed genes (DEGs). Dysregulated expressomes were verified at transcriptional and translational levels by analyzing the GSE49925 dataset and our own samples, respectively. A total of 91 DEGs were identified in the discovery phase, consisting of 15 downregulated genes and 76 upregulated genes. Two hub modules consisting of 12 hub genes were identified. In the verification phase, six of the 12 hub genes exhibited the same variation patterns at the transcriptional level in the GSE49925 dataset. Among them, S100A12 was shown to have the best discriminative performance for predicting in-hospital mortality and to be the only independent predictor of death during follow-up. Validation of 223 samples from our center showed that S100A12 protein level in plasma was significantly lower among patients who survived to discharge, but it was not an independent predictor of survival to discharge or recurrent major adverse cardiovascular events after discharge. In conclusion, the dysregulated expression of plasma S100A12 at the transcriptional level is a robust early prognostic factor in patients with STEMI, while the discrimination power of the protein level in plasma needs to be further verified by large-scale, prospective, international, multicenter studies.

## Introduction

ST-segment elevated myocardial infarction (STEMI) is the most acute manifestation of coronary artery disease (CAD) and is associated with substantial morbidity and mortality (1). Owing to the complexity of the interactions between genetic factors and epigenetic regulatory mechanisms, established cardiovascular risk factors, such as hypertension, diabetes, and hyperlipidemia, are necessary, but not sufficient to fully explain pathogenesis and/or predict the prognosis of the disease (2). Serum biomarkers such as troponin, hypersensitive C-reactive protein, and inflammatory factors play a substantial role in this regard, but nevertheless have a limited value in terms of predictive capacity for diagnosis of early CAD and its prognosis (3).

With the rapid development of new technologies such as next-generation sequencing, great progress has been made in the exploration of diagnostic and therapeutic biomarkers in cardiovascular diseases. Bioinformatics analysis of these “big data” provides novel clues and core data for identifying reliable and functional differentially expressed genes (DEGs) and non-coding transcripts (4); nevertheless, reanalyzing these data using an online database remains necessary because this might provide novel insights that were not previously the focus of attention. Moreover, the integrated studies originating from various medical sources save resources and provide evidence for mapping molecular pathogenesis networks of disease.

In this study, we analyzed two publicly available microarray datasets retrieved from Gene Expression Omnibus (GEO), a database repository that archives, annotates, and freely shares high-throughput functional genomics data submitted by the international research community. We screened out common DEGs associated with STEMI, conducted pathway analysis, constructed protein–protein interaction (PPI) networks as well as transcription factor (TF), and miRNA-mediated transcriptional regulation networks using computational approaches. Dysregulated expressomes were validated by another GEO dataset that has relative complete clinical information and the plasma samples of STEMI patients from our center at transcriptional and translational levels, respectively. Based on the bioinformatics analyses and the double validation, our study is expected to provide novel insights into the pathogenesis of STEMI and/or to identify novel altered transcripts that could be potential early-stage biomarkers for adverse clinical outcomes.

## Materials and Methods

### Expression Profile Dataset Selection

To identify transcriptomes potentially involved in the development and progression of acute myocardial infarction/acute coronary syndrome, several related datasets were mined. First, the datasets of mRNA profiling by array were selected by checking within the datasets registered in the GEO DataSets portal, publicly available at NCBI^1^. In particular, for the selection of the suitable datasets, an advanced search was carried out by inserting the search terms “{(“expression profiling by array” [DataSet Type] and acute myocardial infarction) and “*Homo sapiens*” [porgn:_txid9606]}; “{(“mRNA profiling by array” [DataSetType] and acute coronary syndrome) and “*Homo sapiens*”[porgn:_txid9606]}”. Using this first approach, a list of all acute myocardial infarction/acute coronary syndrome datasets containing mRNA expression levels of human was obtained. Of these datasets, only those that respect the following inclusion and exclusion criteria were selected for subsequent evaluations: Inclusion criteria were as follows: (i) datasets containing whole peripheral blood mRNA expression levels of STEMI; (ii) discovery datasets reporting mRNA expression levels of STEMI patients with primary percutaneous coronary intervention (PCI) and healthy control samples; and (iii) validation datasets containing expression profiles and complete intra-hospital data and follow-up outcome after discharge. Exclusion criteria were as follows: (i) the detection platform was RNA sequencing, not microarray; (ii) datasets containing information about expression of circulating endothelial cells, platelets, or nucleated cells related cell lines (not plasma); and (iii) datasets in which all or some of the patients did not receive primary PCI treatment.

### Data Processing and Differentially Expressed Genes Identification

The raw microarray data of the two datasets downloaded from the GEO database were processed using the online tool GEO2R^2^ to identify genes that are differentially expressed in peripheral blood between healthy controls and patients with STEMI. The specific threshold value for analysis was referred to the previous literature (5). Briefly, the adjusted *P*-values (adj.P) and Benjamini and Hochberg false discovery rate were applied to provide a balance between discovery of statistically significant genes and limitation of false-positives. Probe sets without corresponding gene symbols or genes with more than one probe set were removed or averaged, respectively. (| logFC|) > 1.0 and adj.P < 0.05 were set as the cutoff criteria for DEGs. The lists of the de-regulated genes of the two selected GEO Datasets platforms were subsequently compared to identify the overlapped DEGs shared by two datasets. Venn diagrams were created using the Venn Diagrams software^3^ to display the overlap of DEGs between the two discovery datasets.

### Functional and Pathway Enrichment Analysis

Upregulated and downregulated genes were subjected to Gene Ontology (GO) and Kyoto Encyclopedia of Genes and Genomes (KEGG) pathway analysis using the Database for Annotation, Visualization, and Integrated Discovery (DAVID, version 6.8^4^) software. *P* < 0.05 was considered statistically significant.

### Protein–Protein Interaction Network Construction and Module Analysis

Protein–protein interaction network construction and module analysis was performed as reported previously (5). PPI network was predicted using Search Tool for the Retrieval of Interacting Genes (STRING, version 10.0^5^) online database. In this study, STRING was used to analyze the PPI of DEGs, and an interaction with a combined score of > 0.4 was considered statistically significant. Then, according to the interaction information, the network was constructed and visualized using the Cytoscape software (version 3.4.0^6^). The plug-in Network Analyzer of Cytoscape was used for further analysis, and the topological properties of the PPI network, node degree, were calculated to search for hubgenes from the PPI network. Molecular Complex Detection (MCODE) of Cytoscape was used to screen the significant modules of PPI network with MCODE scores > 5, degree cutoff = 2, node score cutoff = 0.2, max depth = 100, and *k*-score = 2 set as the cutoff parameters. The biological process analysis of hubgenes was performed and visualized using the Biological Networks Gene Oncology tool (BiNGO) (version 3.0.3) plugin of Cytoscape.

### Transcriptional Factors and MicroRNAs Regulatory Network of Hubgenes

The JASPAR database^7^ was used to analyze the TF-gene interactions for the input genes and to assess the effect of the TF on the expression and functional pathways of the hub gene. The TFs of the hubgenes were predicted from this database and a gene-TF regulatory network was constructed and visualized using Cytoscape.

The miRNA-gene regulatory relationships were identified using both experimentally verified and predicted targets of the 12 selected hubgenes. Predicted targets were obtained from TargetScan (version 6.2) and miRDB (version 5.0) databases. To increase the reliability of the results, only the targets appearing in the two databases were retained in this study.

### Verification of Protein Level of Hub Differentially Expressed Genes S100A12 From Our Own Samples

Transcriptional validation through the GSE49925 dataset suggested that S100A12 had the best discriminative performance for predicting in-hospital mortality and was the only independent predictor of death during follow-up. Since S100A12 is a soluble protein that is secreted into the circulation by neutrophils and monocytes, measuring the protein level of S100A12 in peripheral blood has obviously more clinical significance than the level of transcription. Thus, we tested protein level of S100A12 in plasma of patients with STEMI from our biological sample bank and explored its correlation with survival to discharge and recurrent MACE during follow-up. Here, MACE was defined as a composite of hospitalization with an acute coronary syndrome or heart failure diagnosis or cardiovascular death (6) and was identified by using data from our Hospital Discharge Register and telephonic follow-up. Plasma samples from continuative 223 patients with STEMI who underwent primary PCI from June 2014 to December 2015 were collected, and their S100A12 plasma levels were detected by enzyme-linked immunosorbent assay (ELISA). The last follow-up date for all these participants was 31st of January 2021. Cardiovascular death was defined as death due to acute myocardial infarction, ischemic heart disease, cardiac arrest, malignant arrhythmia, heart failure, and ischemic or hemorrhagic stroke. The study has been approved by the ethics committee in our institute and was conducted according to the ethical guidelines of the Declaration of Helsinki.

### Detection of Plasma S100A12 Concentrations by Enzyme-Linked Immunosorbent Assay

In terms of our validation cohort, 3 ml of arterial blood was drawn from the peripheral artery immediately after successful primary PCI, defined as the recovered blood flow of the culprit vessel to Thrombolysis in Myocardial Infarction Grade 3, and preserved in an ethylene diamine tetra acetic acid anticoagulant tube. The blood was then centrifuged immediately at 1,000 × *g* (4°C, 15 min) to separate the plasma and preserved at −80°C until the assay. The concentration of S100A12 was then determined using commercially available ELISA kits (Human S100A12 DuoSet ELISA kit, Catalog # DY1052-05; R&D Systems, Inc., Minneapolis, MN, United States) according to the manufacturer’s instructions. The optical density was read at 450 nm and adjusted at 570 nm with deviation less than 0.01. The assay has a linear range between 10 and 1,000 pg/ml. The intra- and inter-assay variation of the kit was 10 and 12%, respectively. The kit does not determine other S100 proteins.

### Statistical Analyses

The mRNA expression profile data derived from the GEO datasets were already normalized using the GEO2R software. For description and comparisons of baseline demographic, clinical, and laboratory characteristics, the normal distributed quantitative data were expressed as a mean ± standard deviation; the comparisons between two groups were determined using the *t*-test or *t’*-test. Abnormally distributed quantitative data are expressed as median and interquartile ranges and the Mann–Whitney *U*-tests were used for comparisons. The qualitative data were expressed as frequency and composition. The differences in constituent ratio between two groups were compared using the chi-square test or the Fisher’s exact test. All *P* values were two-sided and considered statistically significant if *P* < 0.05. Using median values as the cutoff values for the markers, receiver operating characteristic curve analysis was used to measure their discriminative performances for promising early biomarkers of mortality. Both univariate (unadjusted) and multivariate (adjusted) logistic regression were performed to determine independent clinical factors and biomarkers that were associated with death. The cutoff for the *P*-value of the included variable was set at 0.1. A stepwise variable selection procedure, using backward elimination, was used to identify the strongest independent variables. A multivariate regression analysis was performed *via* a Cox proportional hazard regression model, aiming to identify factors that might independently influence survival. The relationships between survival time and each independent factor were quantified by calculating hazard ratio (HR) and 95% confidence intervals (CIs). All statistical analyses and plotting were performed using SPSS version 23.0 (SPSS Inc., Chicago, IL, United States), Prism 7 (GraphPad Prism7.00, San Diego, CA, United States), and R^8^ (The R Foundation).

## Results

### Selection of Gene Expression Omnibus Datasets About ST-Segment Elevated Myocardial Infarction

We performed a systematic review of GEO datasets concerning patients with STEMI. The search criteria are as follows: “acute myocardial infarction/acute coronary syndrome” as the keywords, “*homo sapiens*” as the research species, and “GEO Datasets” as the data source (search time up to 1 September 2018). As a result, a total of 18 related datasets were retrieved. The platform of one dataset is RNA-seq (GSE65705), while the others are microarray. Among them, six datasets (i.e., GSE29111, GSE60993, GSE61144, GSE34198, GSE49925, and GSE34571) came from the whole transcriptome study with plasma as the sample type, accounting for the largest proportion. Furthermore, GSE29111, GSE34198, and GSE34571 were excluded due to the absence of a healthy control group, the ambiguity of the time point of blood collection, and no patients receiving PCI, respectively. Thus, a total of three datasets (i.e., GSE49925, GSE60993, and GSE61144) were selected. Thanks to Professor Greg Gibson, we have access to the complete medical records and follow-up information of the patients in GSE49925 (7). The dataset was therefore used as a verification set [healthy controls (93 samples) vs. STEMI (61 samples)] and the other two datasets with a relatively small sample size were training sets [GSE60993: healthy controls (seven samples) vs. STEMI (seven samples)] and [GSE61144:healthy controls (ten samples) vs. STEMI (seven samples)]. The details of the datasets selected are summarized in Supplementary Table 1.

### Identification of Differentially Expressed Genes

**FIGURE 1:**
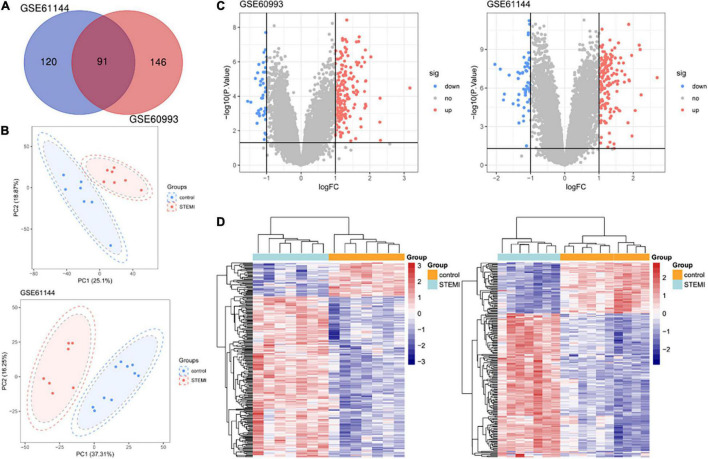
Transcriptome of peripheral blood analysis in GSE61144 and GSE60993 microarray. **(A)** Venn diagram of 91 DEGs from two microarray datasets. **(B)** PCA score plots of STEMI group and healthy control group in these two datasets. **(C)** The volcano plots of DEGs in two datasets. Red indicates genes with high levels of expression, blue indicates genes with low levels of expression, and gray indicates genes with no differential expression based on the criteria of *P* < 0.05 and |logFC| > 1.0, respectively. **(D)** Heatmap of DEGs in two microarrays showed hierarchical clustering of changed transcription of genes in a clustering analysis in different groups. PCA, principal component analysis.

The differential analysis performed by GEO2R on the two gene expression datasets (GSE61144 and GSE60993) allowed the identification of a list of dysregulated genes in patients with STEMI with primary PCI compared with normal healthy controls. As shown in the Venn diagram of DEGs (Figure 1A), a total of 91 common DEGs were obtained, of which 15 were downregulated and 76 were upregulated (Table 1). Next, a principal component analysis model for the DEGs was established for all participants (STEMI and healthy control) in the two discovery datasets. The score plots of their first two principal components are shown in Figure 1B, demonstrating a distinct separation between them on the score plots. The volcanic maps more intuitively showed the distribution of upregulated and downregulated DEGs in STEMI group compared with the control group (Figure 1C). The heatmap of DEGs in these two microarrays showed hierarchical clustering of altered transcription in various groups (Figure 1D) that may facilitate identification of the function of unknown transcripts or the unknown function of known transcripts by collecting similar expression patterns. All these analyses revealed the characteristics and functions of common and special DEG transcripts between the two groups.

**TABLE 1:** Ninety-one DEGs were identified from GSE60993 and GSE61144 microarrays for STEMI.

DEGs	Gene symbol*
Downregulated (15)	***GZMH***, BOLA2, CDC25B, ***CD8A***, CLC, ***GZMA***, KLRG1, EOMES, ***IL2RB***, ADA, ***GZMK***, SBK1,***KLRB1***, ***GNLY***, ***NKG7***
Upregulated (76)	TLR4, RGS2, MGAM, ZDHHC18, NCF4, OPLAH, CEBPD, SLA, ST6GALNAC2, CLEC4D, ABHD5, AQP9, CMTM2, CPD, CSF3R, F5, PGLYRP1, LRG1, CA4, PHC2, PYGL, CD55, ALOX5AP, ***PROK2***, MXD1, ARG1, MANSC1, DUSP1, PADI4, IRAK3, DYSF, CLEC4E, REPS2, TGM3, IL1R2, BMX, FKBP5, ORM1, CAMP, IRS2, ECHDC3, TPST1, ROPN1L, ***S100A12***, TREM1, RBP7, MME, B4GALT5, IL18R1, ***VNN2*,** USP10, QPCT, KCNJ15, ACSL1, CXCL16, CDA, CRISPLD2, SIPA1L2, GAB2, NFIL3, IFRD1, FBXL13, CDK5R1, TSEN34, HMGB2, PANX2, CYP4F3, ***IL18RAP***, ALPL, FOLR3, MCEMP1, CREB5, CKAP4, MMP9, FPR1, VNN

*12 hubgenes (PPI network) are mentioned in bold italics.

*DEGs, differentially expressed genes; STEMI, ST-segment elevated myocardial infarction.*

**FIGURE 2:**
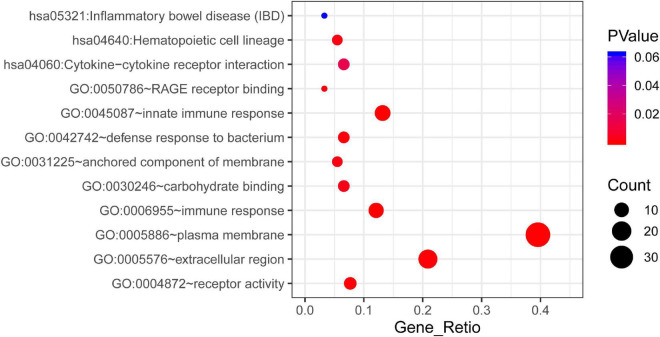
The KEGG pathway and GO enrichment analysis of DEGs in STEMI. The color depth of nodes refers to the corrected *P*-value of ontologies. The size of nodes refers to the numbers of genes that are involved in the ontologies. *P* < 0.01 was considered statistically significant.

According to the results of GO analysis results from the DAVID database, the changes in biological processes of DEGs were significantly enriched in “innate immune response,” “immune response,” and “defense response to bacterium”; the changes in molecular function were enriched in “receptor activity,” “Receptor for advanced glycation endproducts (RAGE) receptor binding,” and “carbohydrate binding”; and the changes in cell component of DEGs were enriched in “plasma membrane,” “extracellular region,” and “anchored component of membrane” (Supplementary Table 2). KEGG pathway analysis revealed that the pathways enriched by dysregulated DEGs included “inflammatory bowel disease,” “hematopoietic cell lineage,” and “cytokine-cytokine receptor interaction pathways.” The top three GO terms and all three KEGG pathways are displayed in a bubble plot visually (Figure 2).

### Protein–Protein Interaction Network Analysis

**FIGURE 3:**
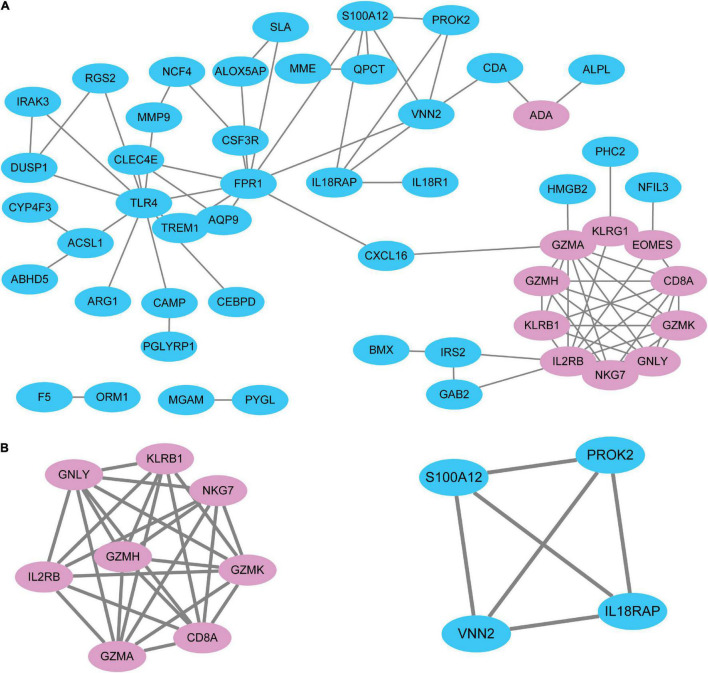
The PPI network of DEGs identified in STEMI. **(A)** A network panorama composed of all the DEGs. Blue nodes indicate upregulated genes, while pink nodes indicate downregulated genes. The lines between nodes represent the interactions between genes. **(B)** Two key modules were identified based on MCODE (molecular complex detection) analysis. Left: Cluster 1. Right: Cluster 2. The biological process analysis of hubgenes from Cluster 1 (Supplementary Figure 1) and Cluster 2 (Supplementary Figure 2) was constructed using BiNGO.

To systematically analyze biologic functions of the obtained DEGs between the two groups, the PPI network of DEGs was constructed using Cytoscape with protein interaction information. With a PPI score > 0.4, a PPI network with 51 nodes and 77 edges was constructed (Figure 3A). Two modules consisting of twelve hubgenes were obtained from a PPI network of DEGs using MCODE (Figure 3B), one with eight nodes and 27 edges (Cluster 1), and the other with four nodes and six edges (Cluster 2). The biological process analysis of the hubgenes is shown in Supplementary Figure 1 (Cluster 1) and Supplementary Figure 2 (Cluster 2), mainly related to “cytotoxic T cell differentiation,” “cleavage of cytoskeletal proteins involved in apoptosis,” “cleavage of lamin,” “pantothenate metabolic process,” “response to wounding,” “inflammatory response,” and “defense response.”

### Hub Gene–Transcription Factor and miRNAs Regulatory Network Analysis

For the 12 hubgenes we identified, a gene-TF regulatory network was constructed including 12 genes and 206 TFs (Supplementary Figure 3). A series of key TFs, such as ZNF263, ZNF384, KLF5, SP1, and SP3, was identified with close interactions with more than one hub gene from the gene-TF regulatory network. For example, ZNF263 regulates eight hubgenes; ZNF384, KLF5, SP1, SP3 regulate six hubgenes; and SP2 and RREB1 regulate five hubgenes. In addition, some hubgenes were found to be regulated by more than one TF; for example, hubgene *VNN2* was found to be potentially regulated by HNF1A, HNF4A, HNF4G, HOXA10, HOXB13, IRF1, and IRF3; and the hubgene *S100A12* might be potentially regulated by MEOX1, NFE2, NR2F1, RREB1, SIX1, and SOX3.

Similarly, a visualized gene-miRNAs regulatory network was also constructed using Cytoscape, including nine hubgenes and 201 miRNAs, Here, no positive results were obtained for GZMH, GZMA, and KLRB1 after intersecting the predicted results of miRNAs from the two databases. As a result, a series of key *Homo sapiens* miRNAs, including *has-miR-4419a*, *has-miR-5001-5p*, *has-miR-4498*, *has-miR-6836-5p*, and *has-miR-6132*, were demonstrated to interact with more than one hubgene from the regulatory network (Supplementary Figure 4).

### Clinical Parameters and 12 Hub Differentially Expressed Genes Expression in Validation Dataset GSE49925

A total of 338 subjects were recruited in GSE49925, including 93 healthy controls, 61 patients with STEMI, 91 patients with CAD, and 93 patients with old myocardial infarctions. According to the original data provided by the initial authors (7), we compared the demographic and clinical characteristics between the healthy controls and STEMI patients (Supplementary Table 3) and validated the expression alterations of 12 hub DEGs that were screened from the discovery datasets. As shown in Supplementary Figure 5, of the 12 hubgenes screened out in the discovery datasets, six exhibited the same variation patterns in GSE49925. Compared with healthy controls, the expression of *IL2RB* and *NKG7* was significantly downregulated, while the expression of *IL18RAP*, *PROK2*, *S100A12*, *VNN2* was significantly upregulated in patients with STEMI.

### Univariate and Multivariate Analysis of Cardiac Death During Follow-Up in the GSE49925 Dataset

According to the GSE49925 original data provided by the author (7), the participants were followed for a mean of 2.4 years to monitor for cardiovascular death. Among 61 patients with STEMI, 55 patients survived and six suffered cardiac death during follow-up. The univariate analysis of cardiac death during follow-up was conducted and shown in Supplementary Table 4 (clinical characteristics) and Supplementary Table 5 (DEGs), respectively. After comparing the transcription profile of 12 hub DEGs in the two groups, we found that original *NKG7* and *S100A12* expression levels were significantly different in sub-groups with different outcomes; *NKG7* levels were significantly lower, while *S100A12* levels were significantly higher in the death group during follow-up. Furthermore, a trend of significant difference was found in terms of the comparison of IL2RB level. Thus, the three indicators of NKG7, S100A12, and IL2RB were included in multivariate Cox regression analysis, in which *S100A12* was further confirmed to be a unique independent marker of death for the STEMI population during follow-up, with a value of HR of 4.621 (95% CI 1.135–18.816, *P* = 0.033) (Supplementary Table 6).

### Discriminative Performance of the Early Biomarkers to Mortality

We determined the discriminative performance of NKG7, S100A12, and IL2RB, the top three DEGs in the univariate analysis, for predicting mortality after STEMI onset by performing receiver operating characteristic statistics. Overall, based on the median values as the cutoff value for the markers, we found that S100A12 showed the best discriminative performance, with an area under the curve of 0.768 (95% CI, 0.588–0.949; *P* = 0.032), and the sensitivity and specificity were 83.3 and 69.1%, respectively (Supplementary Table 7).

### Predictive Value of Plasma S100A12 Protein Level for Prognosis

The baseline characteristics of our validation cohort are presented in Table 2. The patients who died in hospital had significantly faster heart rate, lower systolic blood pressure at admission, worse cardiac function, a higher prevalence of cardiopulmonary resuscitation before reperfusion, and usage of a mechanical circulation support device. It is worth noting that the protein level of S100A12 in plasma was not an independent predictive factor to intra-hospital mortality, although it was significantly higher in the death group than that of the survival group (Table 3).

**TABLE 2:** Univariate analysis of survival to discharge in our validated cohort.

Characteristics	All patients	Survival	Death	Statistics	*P value*
	(*n* = 223)	(*n* = 213)	(*n* = 10)	(*t/Z/*χ*^2^*)	
Age (years)	63.0 ± 11.8	63.2 ± 11.4	59.2 ± 18.3	–0.685	0.510
Male gender, *n*(%)	166(74.4)	158(74.2)	8(80)	0.170	1.000
BMI (kg/m^2^), *n*(%)	24.9 ± 3.7	24.9 ± 3.7	25.3 ± 4.4	0.302	0.763
Hypertension, *n*(%)	121(54.3)	117(54.9)	4(40)	0.858	0.518
Diabetes, *n*(%)	51(62.9)	50(23.5)	1(10)	0.983	0.461
CAD, *n*(%)	49(22.0)	47(22.1)	2(20)	0.024	1.000
Killip III/IV	16(7.2)	10(4.7)	6(60)	43.866	< 0.001
S2D (hours)	3.0(1.5,6.0)	3.0(1.5,6.0)	3.0(2.0,5.3)	0.015	0.988
Heart rate (bpm)	75.8 ± 18.2	77.3 ± 17.3	96.4 ± 26.3	2.269	0.048
SBP (mmHg)	135.0 ± 23.9	136.7 ± 22.8	98.0 ± 17.7	–5.296	< 0.001
Emergency PCI, *n*(%)	176(78.9)	167(78.4)	9(90)	0.772	0.692
Gensini score	55(37, 82)	54(36, 82)	75(39,112)	–1.119	0.263
Culprit vessel, *n*(%)					
LAD/LM	105(47.1)	98(46.0)	7(70)	2.206	0.196
CPR before reperfusion	19(8.5)	12(5.6)	7(70)	50.771	< 0.001
IABP or ECMO use	12(5.4)	6(2.8)	6(60)	61.342	< 0.001
LVEF (%)	49(45,53)	50(46,53)	37(25,48)	–3.238	0.001
S100A12	17.4(9.3,36.0)	15.9(9.3,33.9)	70.9(33.3,530.8)	–3.641	< 0.001
BNP (pg/ml)	69(23,197)	69(23,189)	143(13,304)	–0.371	0.711
CKMB U/L)	137(62, 239)	129(62, 233)	431(92,818)	–2.653	0.008

*BMI, body mass index; BNP, brain natriuretic peptide; CAD, coronary artery disease; CKMB, creatine kinase MB subtype; CPR, cardiopulmonary resuscitation; ECMO, extracorporeal membrane oxygenation; IABP, intra-aortic balloon pulsation; LVEF, left ventricular ejection fraction; PCI, percutaneous coronary intervention; SBP, systolic blood pressure; STEMI, ST-segment elevated myocardial infarction; S2D, the duration from symptom onset to door.*

**TABLE 3:** A univariate and multivariate logistic regression model evaluating the association of clinical factors with in-hospital mortality in our validated cohort.

Variables	HR	HR (95%*CI*)	*P*-value
**Univariate**			
Killip III/IV	30.450	7.393–125.411	< 0.001
Heart rate > 94 bpm	9.911	2.631–37.327	0.001
SBP < 126 mmHg			0.995
CPR before reperfusion	39.083	8.963–170.422	< 0.001
IABP or ECMO use	51.750	11.511–232.655	< 0.001
LVEF > 40%	0.044	0.011–0.185	< 0.001
S100A12 > 36 ng/ml	14.043	2.884–68.382	0.001
CKMB > 392 U/L	17.294	4.444–67.296	< 0.001
**Multivariate**			
LVEF > 40%	0.100	0.018–0.538	0.007
IABP or ECMO use	11.875	2.070–68.128	0.006

*CPR, cardiopulmonary resuscitation; ECMO, extracorporeal membrane oxygenation; IABP, intra-aortic balloon pulsation; LVEF, left ventricular ejection fraction; SBP, systolic blood pressure.*

Next, the prognostic value of the variants to major adverse cardiovascular events (MACE) after discharge was evaluated. The median follow-up time was 76 months (interquartile range 73–79) and a total of 79 patients developed the MACE. It was found that the patients with recurrent MACE had a higher prevalence of diabetes, a higher Gensini score, and lower left ventricular ejection fraction compared with the no-MACE group, while no significant difference was observed in the level of S100A12 between the two groups (Table 4). Spearman correlation analyses showed that the level of S100A12 had a significant correlation with the peak value of creatine kinase MB isoenzyme (CKMB) (*r* = 0.165, *P* = 0.014), but not with Gensini score. Multivariate Cox proportional hazard analyses revealed that Gensini score, rather than S100A12 level, was the only independent prognostic factor to the MACE (HR 3.346, *P* = 0.001) during follow-up after adjustment for age, sex, the time from the onset of symptoms to the visit, and mechanical circulatory support (Table 5).

**TABLE 4:** Clinical characteristics of patients with MACE during follow-up in our validated cohort.

Characteristics	All patients (*n* = 213)	MACE (*n* = 79)	No MACE (*n* = 134)	Statistics (*t/Z/*χ*^2^*)	*P-value*
Age (years)	63.2 ± 11.4	62.2 ± 11.4	64.8 ± 11.3	–1.597	0.112
Male gender, *n*(%)	158(74.2)	57(72.2)	101(75.4)	0.269	0.604
BMI (kg/m^2^), *n*(%)	24.9 ± 3.7	23.0 ± 3.7	24.6 ± 3.7	0.731	0.465
Hypertension, *n*(%)	117(54.9)	41(51.9)	76(56.7)	0.466	0.495
Diabetes, *n*(%)	50(23.5)	26(32.9)	24(17.9)	6.226	0.013
CAD, *n*(%)	47(22.1)	22(27.8)	25(18.7)	2.442	0.118
Killip III/IV	10(4.7)	5(6.3)	5(3.7)	0.750	0.505
S2D (hours)	3(1.5,6)	3(1.5,6.0)	3(1.5,6.0)	0.658	0.510
Heart rate (bpm)	77.3 ± 17.3	76.9 ± 16.0	78.1 ± 19.5	–0.463	0.644
SBP (mmHg)	136.7 ± 22.8	137.6 ± 22.7	135.2 ± 23.0	0.765	0.445
Emergency PCI, *n*(%)	167	62	105	< 0.001	0.983
Gensini score	54(36,82)	60(42,98)	52(34,77)	2.302	0.021
Culprit vessel					
LAD/LM, *n*(%)	98(46.0)	41(51.9)	57(42.5)	1.753	0.185
CPR before reperfusion	12(5.6)	4(5.1)	8(6.0)	0.077	1.000
IABP or ECMO use	6(2.8)	3(3.8)	3(2.2)	0.441	0.673
LVEF (%)	50(46,53)	49(45,53)	50(46,54)	–2.073	0.038
S100A12 (ng/ml)	15.9(9.3,33.9)	18.1(8.3,34.0	14.6(9.4,83.5)	–0.438	0.661
BNP (pg/ml)	69.1(23.4,189.1)	65.1(23.1,161.6)	69.8(23.6,318.0)	1.376	0.169
CKMB U/L)	129(62,233)	122(45,226)	149(74,239)	1.158	0.247

*BMI, body mass index; BNP, brain natriuretic peptide; CAD, coronary artery disease; CKMB, creatine kinase MB isoenzyme; ECMO, extracorporeal membrane oxygenation; IABP, intra-aortic balloon pulsation; LAD, left anterior descending artery; LM, left main artery; LVEF, left ventricular ejection fraction; SBP, systolic blood pressure; S2D, the duration from symptom onset to door.*

**TABLE 5:** A multivariate Cox regression model evaluating the association of clinical factors with MACE during follow-up in our validated cohort.

Variables	HR	95%*CI*	*P*-value
**Univariate**			
Gensini score*	3.346	1.695–6.606	0.001
LVEF*	0.521	0.292–0.929	0.027
Diabetes	2.248	1.180–4.283	0.014
**Multivariate**			
Gensini score	3.346	1.695–6.606	0.001

*The cutoff based on ROC analysis: LVEF 50%, Gensini score 85.

*HR, hazard ratio; CI, confidence interval; LVEF, left ventricular ejection fraction.*

## Discussion

Gene expression profiling by microarray is a comprehensive tool to elucidate underlying mechanisms of disease and to identify disease-related genes as well as dysregulated pathways that may not have been previously linked to cardiovascular diseases (8). Nevertheless, the huge amount of data, either derived from individual basic science experiments, or collected by large international consortia, often generate conflicting results. This discrepancy could be attributed to the varying sample sizes, different ethnic groups involved, and inconsistent disease states of recruited patients. Under such circumstances, integration of various transcriptome profiling datasets could be a promising way to eliminate bias and locate reliable biomarkers for disease.

To evaluate the sensitivity and feasibility of different gene expression profiling of peripheral blood, first, we conducted a genome-wide microarray analysis of the whole blood of two carefully selected discovery cohorts of healthy controls and patients with STEMI. Our analysis revealed 91 DEGs, consisting of 15 downregulated genes and 76 upregulated genes that were functionally enriched for inflammatory disease, immune response, and cytokine receptor interaction networks and pathway in patients with STEMI compared with controls. These findings are consistent with the previous reports, which are based on *in vivo* models of ischemia and reperfusion injury that also demonstrated expression profiles enriched in relative pathways, raising the possibility that transcriptional variation in related pathways contributed to the myocardial pathological progression (9).

We selected 12 DEGs as critical hubgenes in the PPI network with degree ≥ 10. The transcriptional levels of these DEGs were further verified in the validation dataset GSE49925; six genes exhibited identical expression variation patterns in both discovery datasets and validation dataset; compared with healthy controls, IL2RB and NKG7 were significantly downregulated, while IL18RAP, PROK2, S100A12, and VNN2 were significantly upregulated in patients with STEMI.

*IL2RB*, the interleukin 2 receptor beta, is involved in receptor-mediated endocytosis and transduction of mitogenic signals from interleukin 2. Campanella et al. found that the methylation of two loci in *IL2RB* gene was associated with obesity-related disease (10). Transcriptional profiling of peripheral blood cells revealed that the *IL2RB* pathway was involved in Th2 polarization in early systemic sclerosis, a rare condition related to proliferation of extracellular matrix, immune activation, and endothelial cell dysfunction (11). *NKG7* (Natural Killer Cell group 7) was identified as being differentially expressed in human Tregs and T conventional cells (12). Upregulation was previously suggested as an important role for macrophages in renal allografts and intestinal transplantation rejection (13, 14); downregulation in peripheral blood mononuclear cells was reported to be associated with enthesitis-related arthritis (15). Macrophages, T cells, natural killer cells, and peripheral blood mononuclear cells were ascribed key roles in atherosclerotic plaque rupture (16). Downregulated *IL2RB* and *NKG7* raise the possibility that immunology transcriptional changes in circulating cells may reflect myocardial pathological changes. Notably, sub-group analysis of patients with STEMI with different outcomes at 2.4-year follow-up was sensitive for distinguishing *NKG7* expression; its expression levels were significantly lower in patients with STEMI who died; however, as an independent predictor of mortality, *NKG7* had only moderate sensitivity. Further studies with larger sample sizes are needed to confirm its prognostic value.

In the validation phase, another four hubgenes (i.e., *IL18RAP*, *PROK2*, *VNN2*, and *S100A12*) were found to be upregulated in patients with STEMI. *S100A12* was shown to be correlated with the onset of acute coronary syndrome (17) and serves as a promising prognostic biomarker for adverse events in patients with heart failure (18). IL18RAP is an accessory subunit of heterodimeric receptor for interleukin-18 that enhances IL18-binding activity and is involved in the IL-18 pathway, affecting disease risk. Increased IL-18 expression has been localized in human atherosclerotic plaques and was associated with plaque instability (18). Animal models supported the role of interleukin-18 in the development of atherosclerotic lesion and plaque vulnerability (19, 20) as well as the beneficial effect of inhibiting interleukin-18 on plaque progression and composition (21).

PROK2 (prokineticin-2), a member of the endocrine gland derived VEGF family, enhanced angiogenesis and functional recovery from hind limb ischemia and may act as a “biological capacitor” to relay and sustain the pro-angiogenic effect of VEGF (22). In cardiomyocytes, PROK2-PROK receptor 1 (PROKR1) protects cardiomyocytes against hypoxia-induced apoptosis (23). Transient PROKR1 gene transfer reduced mortality and preserved left ventricular function by promoting angiogenesis and cardiomyocyte survival in a coronary ligation mouse model of myocardial infarction (24).

Literature retrieval results uncovered that the interaction between hubgenes (*VNN2*, *S100A12*) and acute myocardial infarction has not been well established previously. *VNN2* has been shown to be expressed in a subset of CD14^+^ monocytic cells, expressing CD11b, CD32, and CD64 with superior phagocytosis, reduced antigen presentation, and high reactive oxygen species production (25). VNN2 facilitates CD15^+^ neutrophil transendothelial migration (26) and possesses pantetheinase activity, being involved in oxidative and pro-inflammatory processes (27, 28).

S100A12 is a proinflammatory cytokine-like protein belonging to the S100 family of calcium-binding proteins (29). S100 proteins mainly function as calcium-dependent activated sensor proteins (30). S100A12 is mainly expressed in myeloid cells and is related to innate immune function. After the cells are stimulated by infection, autoimmune injury, and inflammation, S100A12 proteins are actively secreted to the circulation by an alternative pathway bypassing the classical Golgi pathway, playing an early warning role of the risk of pro-inflammatory damage (31–33). For instance, S100A12 is upregulated at inflammation sites such as bronchoalveolar lavage fluids of inflamed lungs, colonic mucosa of inflamed bowel, and synovial fluid of inflamed joints, which suggests that S100A12 plays a crucial role in inflammatory process and might be a surrogate of local and systemic inflammation (31, 32).

Accumulating evidence suggests that S100A12 protein levels in peripheral blood are related to the occurrence and progression of atherosclerotic lesions (34). However, most studies were conducted in patients with stable (chronic) coronary heart disease. S100A12 protein levels in peripheral blood have been reported to be an independent factor for predicting MACE in patients with stable CAD *via* multivariate Cox proportional hazard models (35). However, little is known about the role of this biomarker in the diagnosis and prognosis of acute coronary syndrome. The increase of S100A12 protein level in blood, which is related to the fragility and rupture of atherosclerotic plaque, has been demonstrated to be a powerful predictor of the progression of stable coronary heart disease to acute coronary syndrome (17, 36). In a recent multicenter prospective study, the protein level of S100A12 in peripheral blood was found to be a biomarker for early diagnosis and an independent predictor of major cardiovascular adverse events within 1 year after STEMI onset (37). In the present study, we innovatively used a strategy combining bioinformatics analysis and experimental validation to explore the correlation between S100A12 mRNA and protein levels and intra and out-of-hospital outcomes in patients with STEMI. We conducted a systematic review of all relevant GEO datasets currently available. In the discovery phase, we carefully selected two datasets with good homogeneity, which came from the same research team and the same type of detection platform (38). The length of follow-up (median, 76 months) of our cohort is also longer than that of the previously published literature. We found that upregulated S100A12 at transcriptional level was associated with mortality of patients with STEMI following primary PCI from both discovery phase and validation phase. Using a different regression model, the mRNA level of S100A12 in plasma was shown to be an independent risk factor for death of a patient with STEMI in the validation dataset of GSE49925.

In our validation cohort, however, it was not shown that the protein level of S100A12 in plasma was an independent prognostic factor of in-hospital death and MACE during follow-up, although the level of the survival group was significantly higher than that of the death group. The reasons for the inconsistent conclusion in the two verifications may include: (1) S100A12, as an important early inflammatory index (37), could more effectively reflect the early myocardial inflammatory injury after STEMI onset, while in-hospital and long-term outcomes might be largely affected by multiple confounders, such as door-to-balloon time, intervention strategy (e.g., ischemic postconditioning), and the complexity of coronary artery lesions. In our center, relying on the advantages of extracorporeal life support center for critical disease, administration of extracorporeal membrane oxygenation has become a normal workflow in rescuing patients with cardiopulmonary failure in our center, which has successfully saved a considerable number of dying patients (39, 40) and may thereby affect the predictive value of S100A12 on prognosis to a certain extent. We proposed the following hypotheses that the aggressive and intensive care delivered to critically ill patients can largely affect the prediction ability of S100A12 on the outcome of overall patients. (2) The samples detected by the two validation cohorts were different (mRNA vs. protein). The difference between the two levels may lead to inconsistent conclusions. (3) The heterogeneity in sample size, demographic characteristics, and treatment strategies between the two cohorts. (4) The detection of protein level is easy to be disturbed, with an inferior sensitivity to that of nucleic acid, and our samples inevitably undergo a certain degree of degradation after long-term storage to minimize the detection bias, a hypersensitive human S100A12 ELISA kit was utilized for testing. (5) Racial difference may also be another potential cause, considering that our validation cohort is Chinese and the patients included in the GSE49925 dataset were predominantly Caucasian and African-American. Nevertheless, these possible explanations need to be clarified by further well-designed large-scale, prospective, and international multicenter studies.

This study has some limitations. First, the strict predefined inclusion and exclusion criteria are a double-edged sword. Although it ensures that the datasets included in the discovery stage have good homogeneity, the total number of samples is relatively small, which may limit the discovery of differential genes. Second, there are certain and even significant differences between the two validation cohorts in some baseline characteristics and perioperative treatment strategies, which has brought some inference to the comparative analysis of the results. Of particular note, the plasma samples in our validation cohort were collected immediately after completion of emergency PCI procedure, while the collection time point of the training datasets and the validation dataset GSE49925 was immediately before emergency PCI. The combination of time and intervention factors can have a complex effect on plasma S100A12 protein levels and may be one of the potential reasons for the differences between our validation results and the one from GSE49925. Thus, the conclusions of this study should be interpreted with caution, and it is necessary to carry out more rigorous research for further validation in the future.

## Conclusion

We performed a bioinformatics analysis of whole transcriptome microarray analysis of peripheral blood from patients with STEMI treated with primary PCI. In the discovery phase, we determined the identity of DEGs, their functional roles involved, and the potential regulatory networks through systematically retrieving, screening, and analyzing the publicly available datasets from GEO Datasets by several computational approaches. In the validation phase, we validated them with another publicly published GEO dataset with complete follow-up information, which came from patients of different races from the discovery set and confirmed that only S100A12 at the transcriptional level was an independent risk factor for long-term cardiogenic death. Finally, the plasma samples from our biological sample bank were used to validate the correlation between S100A12 protein level and prognosis. Taken together, this study provides novel insights into identification of prognostic high-risk groups after STEMI. Considering the potential reasons for the inconsistency of prognostic value between transcription level and translation level, well-designed, large-scale, prospective, multicenter studies are warranted to perform.

## Data Availability Statement

The datasets generated and/or analyzed during the current study are available in the GEO Datasets [GSE61144 (https://www.ncbi.nlm.nih.gov/geo/query/acc.cgi?acc=GSE61144), GSE60993 (https://www.ncbi.nlm.nih.gov/geo/query/acc.cgi?acc=GSE60993), and GSE49925 (https://www.ncbi.nlm.nih.gov/geo/query/acc.cgi?acc=GSE49925)]. As open access to individual-level data was not specified in the original application approved by the ethics committee, the underlying data are only available upon reasonable request. Please contact the corresponding authors.

## Ethics Statement

The studies involving human participants were reviewed and approved by the Ethics Committee of Tianjin Third Central Hospital. The patients/participants provided their written informed consent to participate in this study.

## Author Contributions

HZ, LH, YG, CP, and TL contributed to the research design, performance, data analysis, and writing of the study. YL, YW, and BL participated in the PCI of patients with acute myocardial infarction and collection of plasma samples in our center. All authors contributed to the article and approved the submitted version.

## Conflict of Interest

The authors declare that the research was conducted in the absence of any commercial or financial relationships that could be construed as a potential conflict of interest.

## Publisher’s Note

All claims expressed in this article are solely those of the authors and do not necessarily represent those of their affiliated organizations, or those of the publisher, the editors and the reviewers. Any product that may be evaluated in this article, or claim that may be made by its manufacturer, is not guaranteed or endorsed by the publisher.

## Footnotes

1
www.ncbi.nlm.nih.gov/geo/

2
http://www.ncbi.nlm.nih.gov/geo/geo2r

3
http://bioinformatics.psb.ugent.be/webtools/Venn/

4
david.ncifcrf.gov/home.jsp

5
http://string-db.org

6
http://www.cytoscape.org/

7
http://jaspar.genereg.net/

8
http://www.R-project.org

## Supplementary Material

Supplementary Figure 1 The biological process analysis of the hubgenes in Module 1.

Supplementary Figure 2 The biological process analysis of the hubgenes in Module 2.

Supplementary Figure 3 The hub gene-transcription factor (TF) regulatory network. Red nodes represent the hubgenes, and blue triangular nodes represent TFs.

Supplementary Figure 4 The hub gene-miRNAs regulatory network. Red nodes represent the hubgenes, and blue nodes represent miRNAs.

Supplementary Figure 5 Validation of 12 hubgenes in GSE49225. STEMI (ST-segment elevation myocardial infarction, *n* = 61), CTRL (control, *n* = 93).
