# Supplementary material for: Human Plasma Transcriptome Implicates Dysregulated S100A12 Expression: A Strong, Early-Stage Prognostic Factor in ST-Segment Elevated Myocardial Infarction: Bioinformatics Analysis and Experimental Verification
Source: Front Cardiovasc Med. 2022 Jun 1;9:874436. doi: 10.3389/fcvm.2022.874436 (PMC9200219; doi:10.3389/fcvm.2022.874436)
Supplement: Supplementary Figure 1 — The biological process analysis of the hubgenes in Module 1. [file Data_Sheet_1.zip › supplementary files and tables/Supplementary Tables.docx]

Supplementary Table1 Summary of GEO datasets about AMI (Part Ⅰ)

| **Platform No.** | **Study No.** | **Sample size** | **GSE No.** | **Molecular type** | **Study No.** |  | **Sample type** | **Study No.** |
| --- | --- | --- | --- | --- | --- | --- | --- | --- |
| GPL570  (microarray) | 1  2  6 | 99  26  52 | GSE66360  GSE29111  GSE48060 | Transcriptome | 1,3,4,5,6,  7,8,10,11,14 |  | Circulating endothelial cells | 1 |
| GPL11154  (RNA-seq) | 3 | 34 | GSE65705 | mRNA | 2,13 |  | Nucleated cells | 6,13 |
| GPL6884  (microarray) | 4 | 33 | GSE60993 | miRNA | 9,12 |  | Platelet | 3,8,9 |
| GPL6106  (microarray) | 5 | 17 | GSE61144 |  |  |  | Plasma | 2,4,5,10,11,12 |
| GPL6244  (microarray) | 7 | 42 | GSE62646 |  |  |  | Monocyte | 7,14 |
| GPL2895  (microarray) | 8 | 17 | GSE24519 |  |  |  |  |  |
| GPL8227  (microarray) | 9 | 7 | GSE24548 |  |  |  |  |  |
| GPL6102  (microarray) | 10 | 90 | GSE34198 |  |  |  |  |  |
| GPL10558  (microarray) | 11 | 338 | GSE49925 |  |  |  |  |  |
| GPL13264  (microarray) | 12 | 30 | GSE34571 |  |  |  |  |  |
| GPL5175  (microarray) | 13 | 54 | GSE29532 |  |  |  |  |  |
| GPL6883  (microarray) | 14 | 26 | GSE28454 |  |  |  |  |  |

Abbreviation: AMI acute myocardial infarction

Supplementary Table1 Summary of GEO datasets about AMI (Part Ⅱ)

| **Patient Grouping** | **Study No.** |  | **Timing of Blood Collection** | **Study No.** |
| --- | --- | --- | --- | --- |
| AMI vs. Healthy ctrl | 1,3,6,7,8,9,10,11,12,13,14 |  | Pre-PCI | 1 |
| AMI vs. UA | 2 |  | 7 and 30 days after the index event | 2 |
| AMI vs. UA vs. Healthy ctrl | 4,5 |  | Pre- and post PCI immediately | 3 |
| Survival vs. Death in AMI  patients | 11 |  | Before PCI (after insertion of arterial sheath). All patients received PCI within 4 hours after onset of AMI | 4 |
| LV dilation vs. non-dilation in AMI patients | 12 |  | Emergency department, pre-PCI and 7 days after PCI | 5 |
|  |  |  | Within 48 hours after onset of AMI | 6 |
|  |  |  | The first day, 4 to 6 days and 6 months after onset of AMI | 7 |
|  |  |  | Within 6 hours after onset of AMI | 8 |
|  |  |  | Within 6 hours after onset of AMI | 9 |
|  |  |  | Not available | 10 |
|  |  |  | Immediately before PCI, and the first morning after PCI (fasting) | 11 |
|  |  |  | 3 to 4 days after AMI and at follow-up (median 178 days). Half of the patients received thrombolytic therapy and half received medical treatment (no PCI was performed). | 12 |
|  |  |  | Training set: immediately after diagnosis in emergency room, 2h, 12h, 24h, 36h, 48h after recovery of reperfusion. Validation set: within 48 hours after the diagnosis of AMI | 13 |
|  |  |  | 6 hours, 3 days and 90 days after onset of AMI | 14 |

Abbreviation: AMI acute myocardial infarction, LV left ventricle, PCI percutaneous coronary intervention, UA Unstable angina.

Supplementary Table 2 GO and KEGG pathway enrichment analysis of DEGs (Top 3 terms were listed*)

| Term | Description | Count | P Value | FDR |
| --- | --- | --- | --- | --- |
| **Biological Process** | | | | |
| GO:0045087 | innate immune response | 12 | 1.06E-05 | 0.0157 |
| GO:0006955 | immune response | 11 | 5.16E-05 | 0.0761 |
| GO:0042742 | defense response to bacterium | 6 | 8.44E-04 | 1.239 |
| **Cell Components** | | | | |
| GO:0005886 | plasma membrane | 36 | 2.21E-04 | 0.248 |
| GO:0005576 | extracellular region | 19 | 5.92E-04 | 0.663 |
| GO:0031225 | anchored component of membrane | 5 | 2.19E-03 | 2.430 |
| **Molecular Function** | | | | |
| GO:0004872 | receptor activity | 7 | 6.98E-04 | 0.880 |
| GO:0050786 | RAGE receptor binding | 3 | 1.28E-03 | 1.606 |
| GO:0030246 | carbohydrate binding | 6 | 2.80E-03 | 3.490 |
| **KEGG pathway** | | | | |
| hsa04640 | Hematopoietic cell lineage | 5 | 1.99E-03 | 2.218 |
| hsa04060 | Cytokine-cytokine receptor interaction | 6 | 1.48E-02 | 15.415 |
| hsa05321 | Inflammatory bowel disease | 3 | 6.23E-02 | 51.538 |

Abbreviation: DEGs differentially expressed genes, FDR false discovery rate, GO Gene ontology, KEGG Kyoto Encyclopedia of Genes and Genomes, RAGE receptor of advanced glycation endproducts, STEMI ST-segment elevated myocardial infarction. * The GO terms were ranked by the adjusted P value.

Supplementary Table 3. Comparison of clinical parameters between STEMI and healthy control in GSE49225

| **Characteristic** | **Controls (n=93)** | **STEMI patients (n=61)** | ***P* value** |
| --- | --- | --- | --- |
| **Demographics** |  |  |  |
| Age (years) | 62.7±11.5 | 66.0±10.1 | 0.134 |
| Male (%) | 51(54.8) | 48(78.7) | 0.003 |
| BMI (kg/m^2^) | 30.2(25.4,33.2) | 28.2(24.8,33.4) | 0.880 |
| Current smoker (%) | 18(19.4) | 8(13.1) | 0.482 |
| **Risk factors** |  |  |  |
| Hypertension (%) | 67(72.0) | 47(77.0) | 0.488 |
| Diabetes mellitus (%) | 21(22.6) | 30(49.2) | 0.001 |
| Hyperlipidemia (%) | 61(65.6) | 48(78.7) | 0.080 |
| **Clinical parameters** |  |  |  |
| SBP (mmHg) | 139(124,148) | 127(118,145) | 0.110 |
| DBP (mmHg) | 76.8±10.8 | 73.6±11.0 | 0.417 |
| HDL-C (mmol/L) | 49.0(38.3, 58.5) | 34.0(29.0, 42.0) | <0.001 |
| LDL-C (mmol/L) | 94.0(73.3, 123.8) | 83.0(59.5, 105.0) | 0.033 |
| Triglyceride (mg/dL) | 122.5(83.5, 168.5) | 114.0(79.5, 157.5) | 0.753 |
| Cholesterol (mg/dL) | 169.5(145.0, 202.3) | 148.0(121.0, 178.5) | 0.005 |
| Glucose(mg/dL) | 109.5(96.3, 121.8) | 112.0(99.0, 150.5) | 0.080 |
| Creatinine (mg/dL) | 1.00(0.80, 1.20) | 1.10(0.95, 1.20) | 0.002 |
| WBC(×10^9^/L) | 6.75(5.90, 8.68) | 9.30(7.40, 11.15) | <0.001 |

Abbreviation BMI body body mass index, DBP diastolic blood pressure, HDL-C high density lipoprotein cholesterol, LDL-C low density lipoprotein cholesterol, SBP systolic blood pressure, STEMI ST-segment elevated myocardial infarction, WBC white blood cell

Suppl. Table 4 Comparison of clinical parameters in STEMI patients with different outcomes after discharge in GES49225

| **Characteristic** | **Survive (n=55)** | **Death (n=6)** | ***P* value** |
| --- | --- | --- | --- |
| **Demographics** |  |  |  |
| Age (years) | 64.0±10.8 | 71.5±13.7 | 0.120 |
| Male (%) | 44(80.0) | 4(66.7) | 0.599 |
| BMI (kg/m^2^) | 30.4±6.8 | 24.8±6.3 | 0.057 |
| Current smoker (%) | 6(10.9) | 2(33.3) | 0.045 |
| **Risk factors** |  |  |  |
| Hypertension (%) | 42(76.4) | 5(83.3) | 1.000 |
| Diabetes mellitus (%) | 29(52.7) | 1(16.7) | 0.195 |
| Hyperlipidemia (%) | 43(78.2) | 5(83.3) | 1.000 |
| **Clinical parameters** |  |  |  |
| SBP (mmHg) | 133(119, 149) | 123(113, 147) | 0.461 |
| DBP (mmHg) | 74±12 | 79±7 | 0.339 |
| HDL-C (mmol/L) | 34.0(29.0, 41.0) | 33.5(27.0, 76.3) | 0.782 |
| LDL-C (mmol/L) | 88.6±33.7 | 69.0±24.6 | 0.174 |
| Triglyceride (mg/dL) | 124.0(95.0, 205.5) | 84.0(53.3, 126.0) | 0.063 |
| Cholesterol (mg/dL) | 157.9±35.1 | 133.7±44.4 | 0.127 |
| Glucose (mg/dL) | 115.5(99.8, 158.8) | 89.5(67.0, 150.3) | 0.078 |
| Creatinine (mg/dL) | 1.1(0.9, 1.2) | 1.1(1.0, 9.4) | 0.689 |
| WBC (×10^9^/L) | 9.96±3.86 | 8.70±3.27 | 0.448 |
| Gensini Score | 24(13.6, 47.8) | 80.3(18.3, 181.0) | 0.117 |
| **Medication** |  |  |  |
| ACEI/ARB (%) | 31(66.0) | 4(66.7) | 1.000 |
| Aspirin (%) | 45(91.8) | 4(66.7) | 0.123 |
| Beta blocker (%) | 40(80.0) | 5(83.3) | 1.000 |
| Plavix (%) | 32(66.7) | 2(33.3) | 0.179 |

Abbreviation BMI body body mass index, DBP diastolic blood pressure, HDL-C high density lipoprotein cholesterol, LDL-C low density lipoprotein cholesterol, SBP systolic blood pressure, STEMI ST-segment elevated myocardial infarction, WBC white blood cell

Supplementary Table 5 Comparative analysis of differentially expressed genes in GES49225 populations with different follow-up outcomes

| **Characteristic** | **Survive (n=55)** | **Death (n=6)** | ***P* value** |
| --- | --- | --- | --- |
| *IL2RB* | 7.56±0.50 | 7.17±0.43 | 0.074 |
| *NKG7* | 7.04±0.73 | 6.65±0.26 | 0.016 |
| *IL18RAP* | 9.27±0.90 | 9.90±0.90 | 0.108 |
| *S100A12* | 9.64±0.72 | 10.35±0.57 | 0.024 |
| *VNN2* | 7.88±0.86 | 8.14±0.34 | 0.467 |
| *PROK2* | 9.23±0.97 | 9.63±0.54 | 0.332 |
| *CD8A* | 7.43±0.69 | 7.15±0.79 | 0.358 |
| *GZMH* | 9.58±0.57 | 9.33±0.75 | 0.318 |
| *GZMA* | 7.39±0.64 | 7.10±0.41 | 0.271 |
| *GNLY* | 9.45±0.81 | 9.12±0.76 | 0.341 |
| *GZMK* | 7.95±0.78 | 7.87±0.85 | 0.830 |
| *KLRB1* | 8.12±0.73 | 7.65±0.32 | 0.124 |

Suppl. Table 6 Univariate and multivariate Cox proportional hazard regression model for the mortality of STEMI patients during follow-up in GSE49925

| Variables | HR (95%*CI*) | *P* value |
| --- | --- | --- |
| Univariate |  |  |
| BMI | 0.891(0.781-1.018) | 0.089 |
| Current smoker | 3.055(0.949-9.838) | 0.061 |
| Glucose | 0.986(0.960-1.012) | 0.287 |
| Triglyceride | 0.984(0.964-1.006) | 0.147 |
| IL2RB | 0.187(0.037-0.949) | 0.043 |
| NKG7 | 0.387(0.111-1.352) | 0.137 |
| S10012A | 5.196(1.272-21.228) | 0.022 |
| Multivariate |  |  |
| S10012A | 4.621 (1.135-18.816) | 0.033 |

Abbreviation : BMI body mass index

Suppl.Table 7 ROC analysis of serum IL2RB, NKG7 and S100A12 for predicting mortality after STEMI onset in GSE49925

| Variables | AUC | *P* | 95% *CI* | cutoff | Sensitivity (%) | Specificity (%) |
| --- | --- | --- | --- | --- | --- | --- |
| IL2RB | 0.729 | 0.067 | 0.543-0.914 | 7.69 | 40 | 100 |
| NKG7 | 0.665 | 0.187 | 0.531-0.799 | 6.99 | 50.9 | 100 |
| S100A12 | 0.768 | 0.032 | 0.588-0.949 | 10.06 | 83.3 | 69.1 |
| Combination* | 0.794 | 0.019 | 0.631-0.957 | 0.103 | 83.3 | 70.9 |

*: The equation of fit is -9.502-0.351×IL2RB-0.497×NKG7+1.303×S100A12. Abbreviation: AUC area under the curve, CI confidence interval.
