# Supplementary figures and images for: Human Plasma Transcriptome Implicates Dysregulated S100A12 Expression: A Strong, Early-Stage Prognostic Factor in ST-Segment Elevated Myocardial Infarction: Bioinformatics Analysis and Experimental Verification
Source: Front Cardiovasc Med. 2022 Jun 1;9:874436. doi: 10.3389/fcvm.2022.874436 (PMC9200219; doi:10.3389/fcvm.2022.874436)

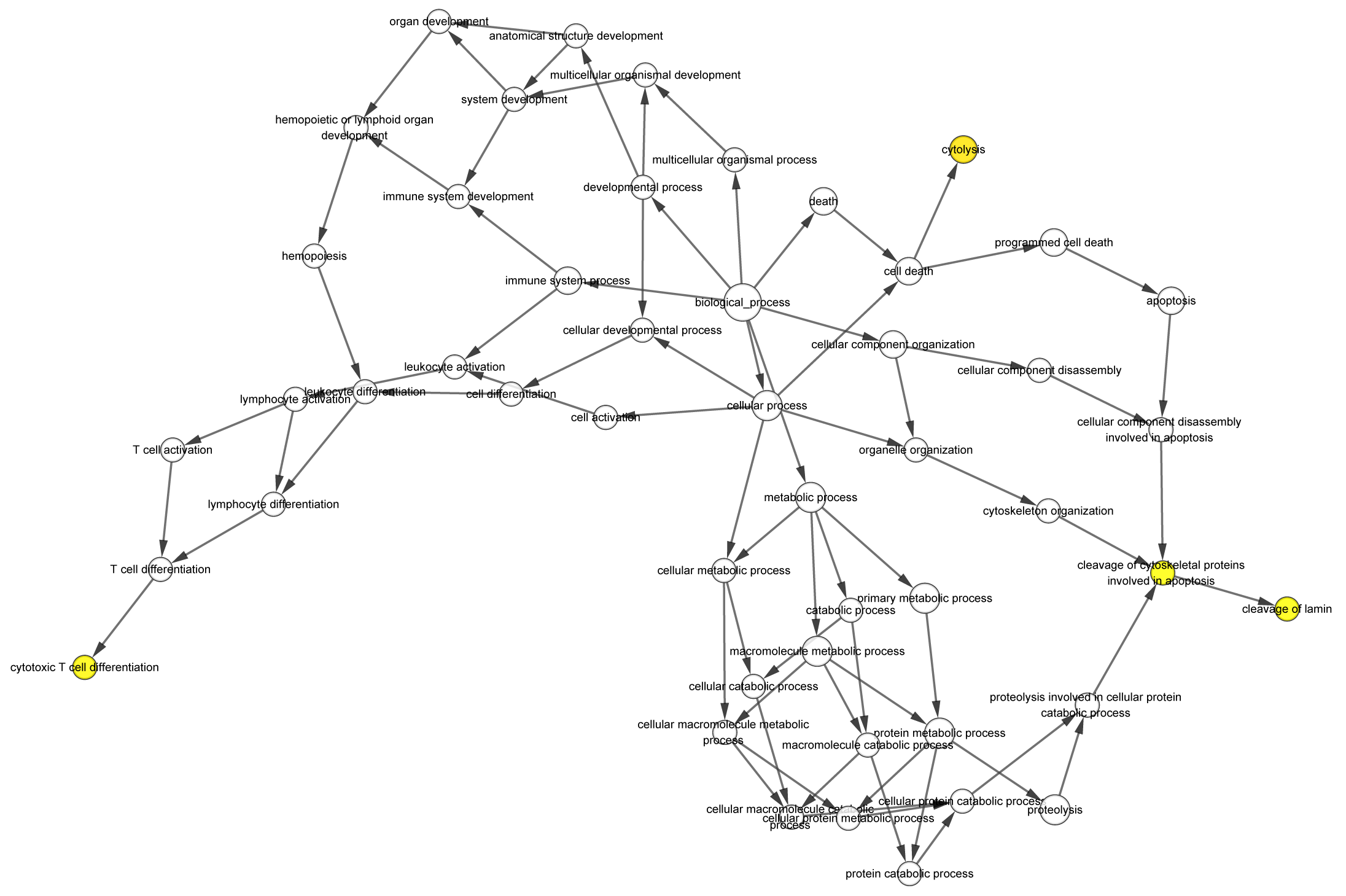

Supplement: Supplementary Figure 1 — The biological process analysis of the hubgenes in Module 1. [file Data_Sheet_1.zip › supplementary files and tables/Supplementary Figure 1.tif]

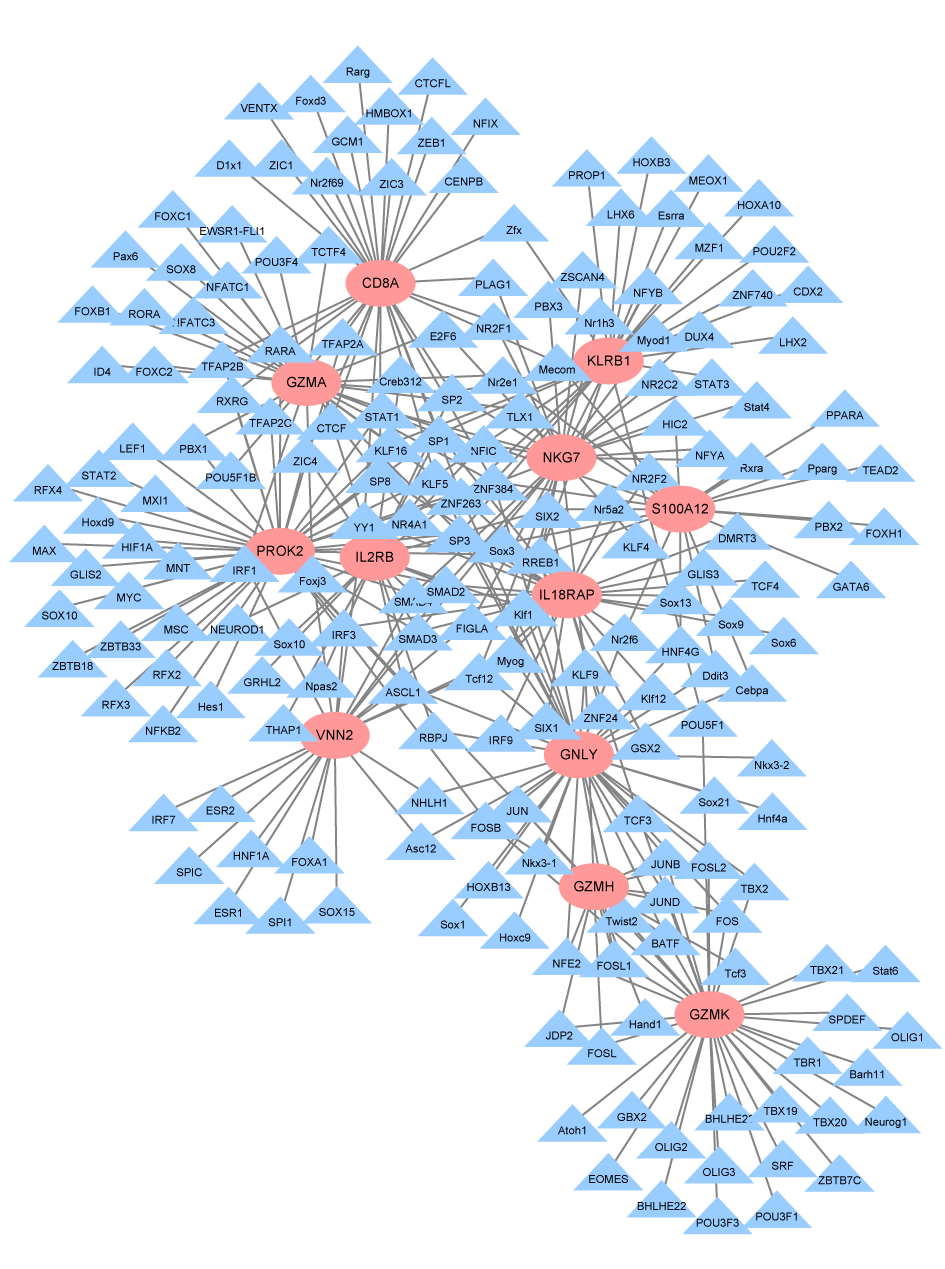

Supplement: Supplementary Figure 1 — The biological process analysis of the hubgenes in Module 1. [file Data_Sheet_1.zip › supplementary files and tables/Supplementary Figure 3.tif]

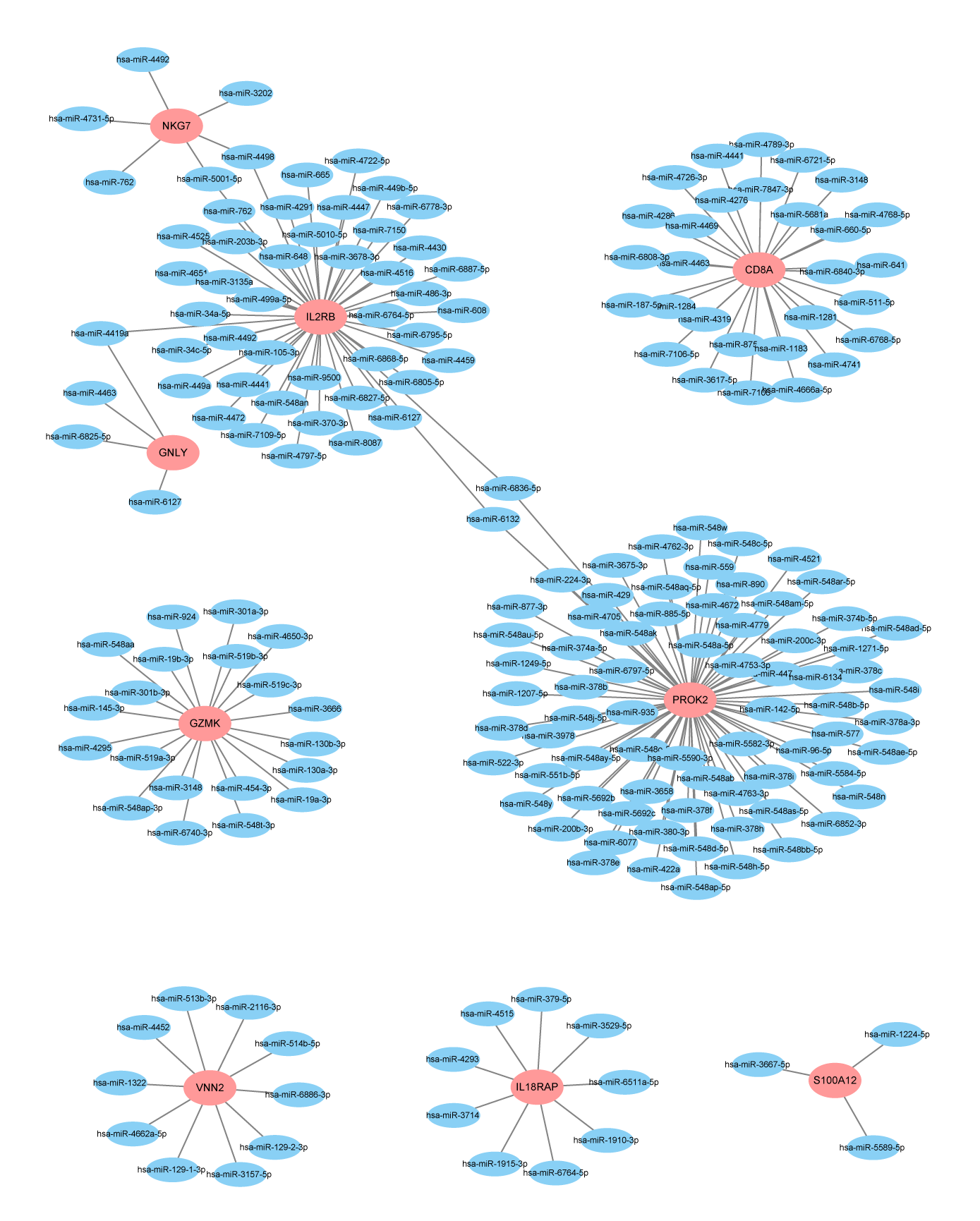

Supplement: Supplementary Figure 1 — The biological process analysis of the hubgenes in Module 1. [file Data_Sheet_1.zip › supplementary files and tables/Supplementary Figure 4.tif]

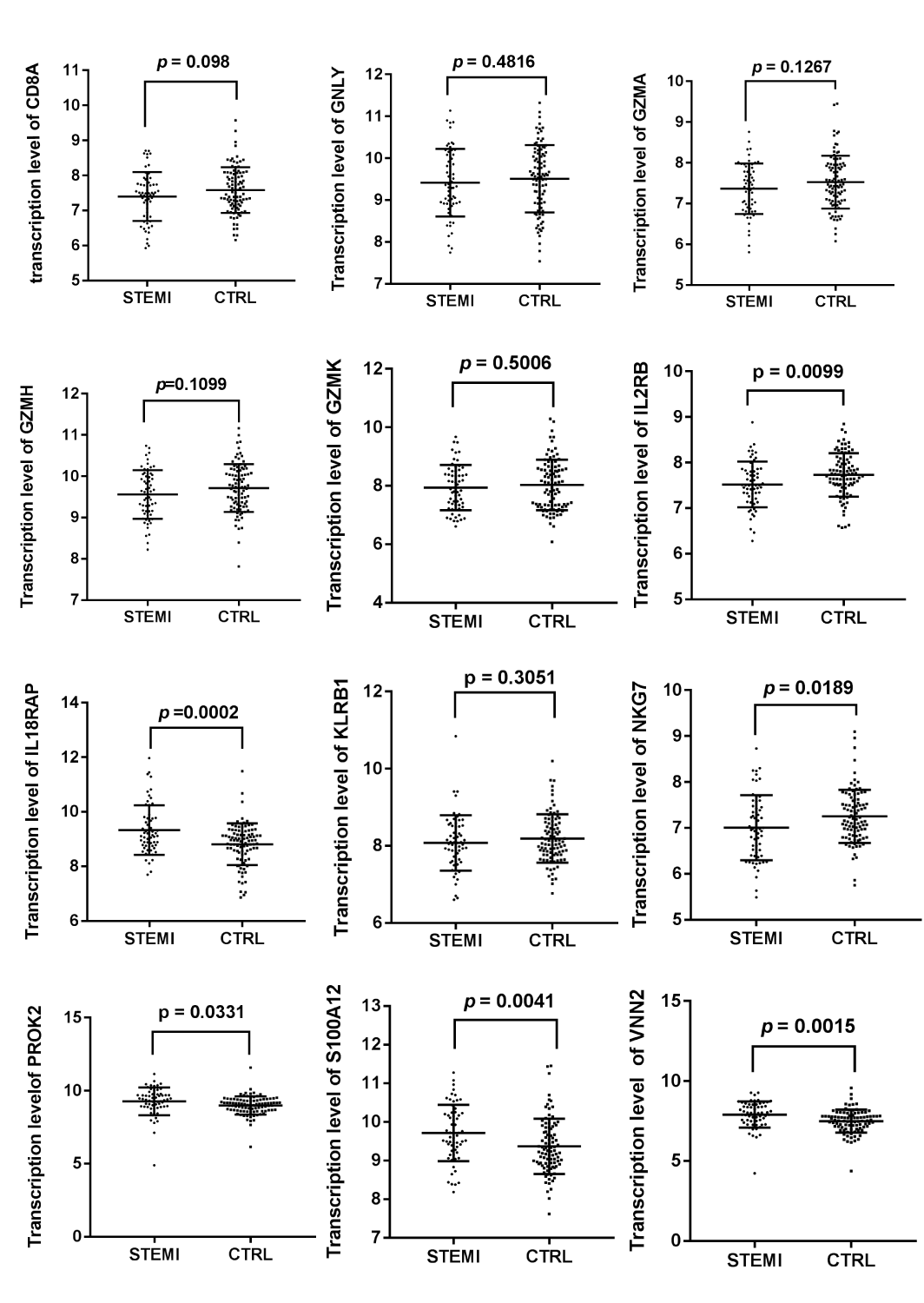

Supplement: Supplementary Figure 1 — The biological process analysis of the hubgenes in Module 1. [file Data_Sheet_1.zip › supplementary files and tables/Supplementary Figure 5.tif]

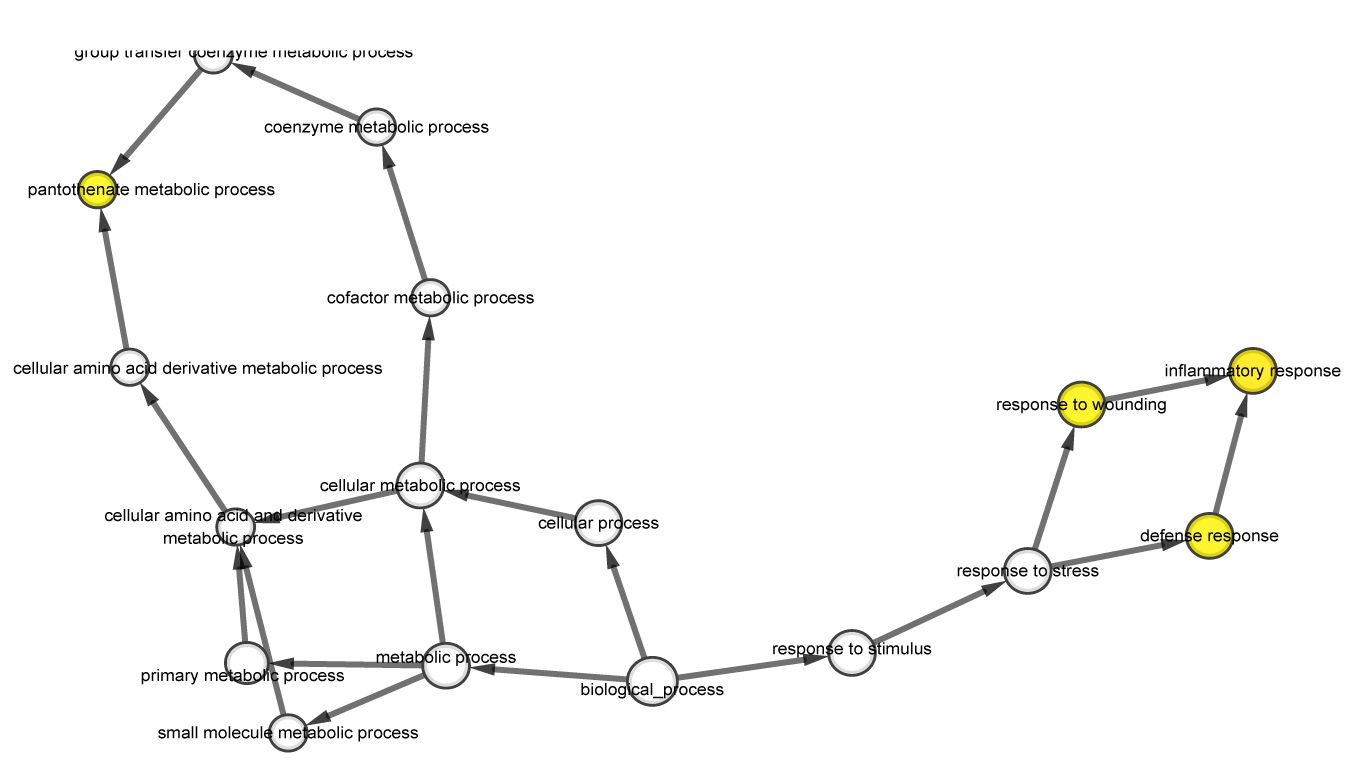

Supplement: Supplementary Figure 1 — The biological process analysis of the hubgenes in Module 1. [file Data_Sheet_1.zip › supplementary files and tables/Supplementary Figure 2.tif]
